# Effect of Blood Flow Restriction on Functional, Physiological and Structural Variables of Muscle in Patients with Chronic Pathologies: A Systematic Review

**DOI:** 10.3390/ijerph19031160

**Published:** 2022-01-20

**Authors:** Álvaro Jesús Reina-Ruiz, Alejandro Galán-Mercant, Guadalupe Molina-Torres, Jose Antonio Merchán-Baeza, Rita Pilar Romero-Galisteo, Manuel González-Sánchez

**Affiliations:** 1 Department of Physiotherapy, Faculty of Health Sciences, University of Málaga, Arquitecto Francisco Peñalosa, 3, 29071 Málaga, Spain; alvaro.reina.ruiz@gmail.com (Á.J.R.-R.); rpromero@uma.es (R.P.R.-G.); mgsa23@uma.es (M.G.-S.); 2 MOVE-IT Research Group, Department of Physical Education, Faculty of Education, Sciences University of Cádiz, 11002 Cádiz, Spain; 3 Biomedical Research and Innovation Institute of Cádiz (INiBICA) Research Unit, Puerta del Mar University Hospital, University of Cádiz, 11002 Cádiz, Spain; 4 Department of Nursing Science, Physiotherapy and Medicine, Faculty of Nursing and Physiotherapy, University of Almeria, 04120 Almeria, Spain; 5 Centre for Health and Social Care Research (CESS), Research Group on Methodology, Methods, Models and Outcomes of Health and Social Sciences (M3O), Faculty of Health Science and Welfare, University of Vic-Central University of Catalonia (UVIC-UCC), C. Sagrada Família, 7, 08500 Vic, Spain; josan.merchan@uvic.cat; 6 Instituto de Investigación Biomédica de Málaga, IBIMA, Calle Doctor Miguel Díaz Recio, 28, 29010 Málaga, Spain

**Keywords:** blood flow restriction, musculoskeletal disease, controlled tourniquet, chronic pathologies, occlusion resistance training

## Abstract

The main objective of this systematic review of the current literature is to analyze the changes that blood flow restriction (BFR) causes in subjects with neuro-musculoskeletal and/or systemic pathologies focusing on the following variables: strength, physiological changes, structural changes and cardiocirculatory variables. The search was carried out in seven databases, including randomized clinical trials in which therapeutic exercise was combined with the blood flow restriction tool in populations with musculoskeletal pathologies. Outcome variables are strength, structural changes, physiological changes and cardiocirculatory variables. Twenty studies were included in the present study. Although there is a lot of heterogeneity between the interventions and evaluation instruments, we observed how the restriction of blood flow presents significant differences in the vast majority of the variables analyzed. In addition, we observed how BFR can become a supplement that provides benefits when performed with low intensity, similar to those obtained through high-intensity muscular efforts. The application of the BFR technique can provide benefits in the short and medium term to increase strength, muscle thickness and cardiovascular endurance, even improving the physiological level of the cardiovascular system. In addition, BFR combined with low-load exercises also achieves benefits comparable to high-intensity exercises without the application of BFR, benefiting patients who are unable to lift high loads.

## 1. Introduction

For decades, blood flow restriction (BFR), originating from Japan, has become one of the most popular techniques in physiotherapy [1]. To perform the BFR technique, a controlled tourniquet is performed, generating a gradual mechanical pressure just below it, affecting blood flow (both arterial and venous) and generating a hypoxia in the restricted area. For the realization of the tourniquet, mechanical cuffs are used, which are inflated and regulate the pressure generated in the compression zone, although nylon or elastic cuffs are also used for the realization of the tourniquet [2,3,4,5]. This zone of compression is usually the proximal region of both the upper and lower limbs. For the application of BFR, different levels of arterial occlusion pressure are used depending on the intended objectives. In this sense, two studies published in recent years have helped to determine the qualitative and quantitative aspects of the application of BFR, both in its application as part of training [4,] and as part of a rehabilitation [3] protocol.

BFR is used in combination with strength exercise—low loads of around 20–40% of the maximum repetition that a subject can carry out are used—or through aerobic exercise at an intensity perceived by the subject as low-to-moderate [6]. Furthermore, BFR uses a series of cuffs that occlude the proximal parts of the extremities in order to cause restrictions in the arterial and venous blood flow, and through this phenomenon obtain the same benefits as high-load exercise [7]. The theories by which these results are supported are based on the metabolic effects induced during the hypoxic state that are created by the cuffs, neuromuscular and hormonal reactions [8]. The first theory is based on the increased production of lactate, reactive oxygen free radicals and nitrogen oxide. This in turn leads to greater protein synthesis and muscle recruitment and less protein destruction [9].

In the field of physiotherapy, BFR is becoming increasingly important since it has sparked interest from its application in training, in which it can be extrapolated to therapeutic exercise [10]. Among the methodologies used by this technique, there is heterogeneity in terms of the results as a consequence of the type of occlusion used, pressure of the arterial occlusion or pressure of the limb occlusion, the type of material used, the width of the cuff, the quantity of applied pressure (pressures between 40% and 80% are generally exerted) and the training method (aerobic or strength) [11,12]. For the application of BFR cuffs, it is acknowledged that the understanding of the comorbidities that the individual may present is important, emphasizing deep vein thrombosis as a contraindication, unless there is medical authorization; and rhabdomyolysis, which appears as a result of overexertion and is known to have a low incidence of 0.07–0.2% [4].

Within BFR, it is known that its application has led to the obtainment of greater gains in muscle mass and strength of subjects seeking to improve their performance such as recovering from an injury in its initial stages [13]. For this reason, BFR is considered an important tool for those patients experiencing problems during recovery, since they may present exacerbated symptoms with high-load exercises, or long-term immobilization that may lead to muscle atrophy, simultaneously fulfilling the goals of maintaining or increasing muscle mass and strength [13,14,15]. In addition, this working method can create improvements in the ability to contract muscles to a greater extent, although it has been proven in healthy adults who practice exercise [16] that its benefits depend entirely on the type of exercise performed [aerobic or strength] and the population practicing it [17]. On the other hand, it is reported that BFR can have short-term direct effects on aerobic capacity [18,19] and on the vascular system, presenting a hypotensive effect and increased heart rate and cardiovascular function [20,21].

The body of evidence regarding BFR has substantially increased over the last two decades, including the evaluation of the performance and, to a lesser extent, muscle hypertrophy in healthy subjects and focusing on the evaluation of other variables [6,22]. The most recent reviews collected information regarding physiological changes such as aerobic capacity and structural changes such as muscle cross-sectional area, which provide more information to the currently consolidated evidence [23,24]. However, as of yet no systematic review has analyzed the structural changes produced by BFR regarding strength, as well as metabolic and cardiovascular changes [14,25,26].

To our knowledge, no review exists that explains how BFR influences strength, physiological and structural changes, as well as cardiocirculatory variables in subjects with pathologies. In accordance with the aforementioned points, the main objective of this systematic review of the current literature will be to analyze the changes caused by BFR in subjects with neuro-musculoskeletal and/or systemic pathologies, focusing on the following variables: strength, physiological changes, structural changes and cardiocirculatory variables.

## 2. Materials and Methods

In this study, a systematic review based on current scientific evidence is carried out using PRISMA guideline.

### 2.1. Search Strategy

During the process of developing the search strategy for this systematic review, information collected from various databases was used: PubMed, Embase, Cochrane, PEDro, CINAHL, Scopus, SPORTDiscus and Trip Medical Database. The selected articles were filtered using the following keywords: kaatsu; ischemic training; blood flow restriction; occlusion resistance training; vascular occlusion; vascular restriction; chronic disease. Boolean indicators AND and OR were combined to perform the search.

### 2.2. Document Selection

Once the search strategy was carried out, the inclusion criteria of studies were established as those randomized clinical trials in which therapeutic exercise was combined with the blood flow restriction tool in populations with musculoskeletal pathologies, and which were published between 1 January 2015 and 21 August 2021.

All studies that had been published in a language other than Spanish, English, Italian, French and Portuguese were established as exclusion criteria. Furthermore, those studies whose score on the PEDro scale was less than 6 were excluded.

#### 2.2.1. Selection Method

Scientific literature searches across the different databases and the selection of the above-mentioned documents—applying the inclusion and exclusion criteria—were carried out in parallel with two blinded researchers. In the event that there was any discrepancy between the authors, the selection of the document was resolved by a third blinded investigator.

#### 2.2.2. Methodological Quality

In relation to the methodological quality assessment of the randomized clinical trials, the PEDro assessment scale was used, which consists of 11 questions, 10 of which (selection criteria; selection randomization: hidden allocation; initial comparability between groups; totality of blinded subjects; all therapists blinded; all evaluators blinded; adequacy of follow-up; analysis with intention to treat; comparison of results between groups; existence of specific measures and variability) can be answered in a dichotomous way using Yes/No, which is awarded depending on the fulfillment of the requirements of the particular point. Regarding the unscored question, this cannot be entered due to external validity influences which do not meet the internal validity requirement [27].

In addition, a distinction was made between studies of high (score greater than or equal to 6 points) and low (score lower than 6 points) methodological quality based on the results obtained from this scale [27].

### 2.3. Result Variables

The following were selected as outcome variables for the subsequent analysis of the studies: strength, structural changes, physiological changes and cardiocirculatory variables. The strength variable is defined as the ability of the muscles to produce a muscular contraction in the absence or against a load with an effort to counteract a force, and this can be evaluated in any of its modalities: isometric, isotonic or isokinetic contraction [28,29]. For structural changes, this variable is understood as the area perpendicular to the muscle fibers compared to the total area of the muscle. In addition, its presence, in a greater quantity, is considered a predictor of greater strength, which is why it has a direct relationship with strength [30,31].

The variable of physiological changes is defined as the amount of physical energy used during any physical activity by any type of person compared to rest [32]. In contrast, a cardiocirculatory variable is understood to be the measurable amount in a blood sample or imaging test of certain substances, cells or molecules present in the bloodstream to detect blood markers [33].

According to the analysis of the results of each study variable, a temporal continuity model was applied in order to typify the information collected and thus be able to homogenize the presentation of each data obtained. Subsequently, the values of the results were relativized to 100% on a scale of 0–100 in order to allow comparison between the different results.

Within the continuity model, the so-called baseline was proposed as the moment of data collection just before the intervention group participants could begin. Subsequently, data collection was divided into four moments: short-term, medium-term, long-term and follow-up period. For the short term, the period between the start of treatment and week 6 is attributed; medium term: from week 6 to week 12; long term: between weeks 12 and 24; finally, for the follow-up period: a period of time after the applied intervention that spans more than 24 weeks.

## 3. Results

**Figure 1:**
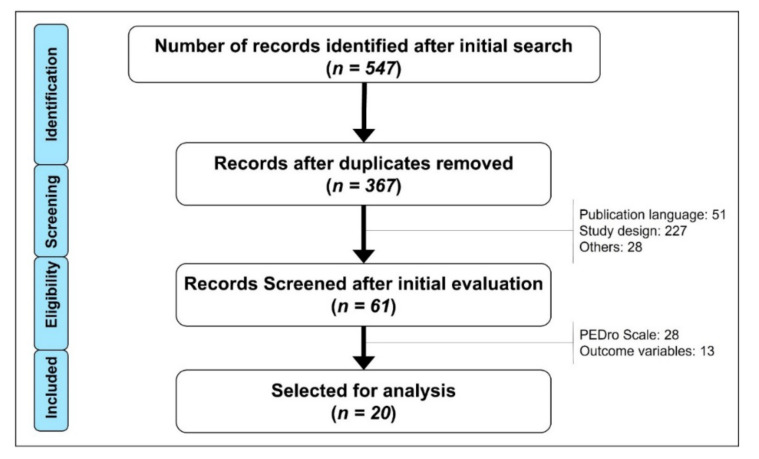
Flow chart of the search, filtering and selection of the included documents.

Figure 1 shows the results of the search. The initial search identified 547 documents; after eliminating duplicate documents, 367 documents were analyzed for an initial evaluation. After the initial evaluation and after a complete reading of the document and analysis of the outcome variables, 20 documents were selected and included in this study. For more details on the document-filtering process, see Figure 1.

Table 1 presents a minimum sample size of 20 patients [34] and a maximum of 79 [35], in which the mean age of the participants was 45 years, ranging from 14 [36] to 76 years [37]. Likewise, when the blood flow restriction cuff was placed, a minimum pressure of 30% of the total blood flow restriction was applied [38] and a maximum of 80% [36]. The interventions used were strength, aerobic type and blood flow restriction training. Regarding the frequency of the sessions, the minimum follow-up was 4 weeks [39,40] and the longest was 16 weeks [35]. Furthermore, the minimum recurrence was two weekly sessions [41] and the maximum was three weekly sessions [42]. The variables evaluated were strength, structural and physiological changes of the muscle and cardiovascular variables at the blood level.

The results of the strength variable are shown in Table 2, using a total of six assessment tools such as the dynamometer [42,44,45], 1 maximum repetition [46,47], 10 maximum repetitions [46], central activation ratio [37], manual muscle test [48] and kinetic communicator [48]. Among all, the dynamometer is the most used, presenting significant heterogeneity as a consequence of the use of different units of measurements and assessment of different anatomical areas, together with the estimation of 1 maximum repetition, where its mean is 112.83 kg, varying between 0.66 kg [37] and 430.6 kg [41]; however, it does not present homogeneity in the evaluation units or the results, due to the variety of exercises used. In addition, all the studies present an intervention duration of up to 6 months and a follow-up of 3 months in a total of five studies.

When it comes to analyzing structural changes in muscle (Table 3), a high heterogeneity is observed in the assessment instruments and, therefore, in the outcome variables. In this sense, imaging techniques are used preferentially, although the outcome variable obtained sometimes allows a comparison between the different studies that use the same technique, but only an approximation to the eventual comparison of the results between the different techniques. Four studies used magnetic resonance imaging, two used computerized tomography and five used ultrasound. However, in the latter instrument, three studies analyzed cross-sectional area while three analyzed muscle thickness (one study, Barbalho et al. [35], analyzed both outcome variables). All imaging techniques are widely used in diagnosis and clinical follow-up; however, at the same time, they all present a series of strengths and weaknesses that must be taken into account when interpreting the results. Table 3 shows structural muscle changes; data were collected from the following four assessment tools: intranuclear magnetic resonance [36,49], ultrasound [37], measuring tape [35,44,50] and computerized tomography [46,47]. Of the different assessment tools, both intranuclear magnetic resonance imaging and ultrasound showed significant heterogeneity between their results as a consequence of using different units of measurement in the data-collection step, as well as the different muscle groups assessed. Computerized tomography presented an average of 4787 mm^2^, with minimum and maximum values of 3825 and 5750 mm^2^, respectively [46], therefore presenting homogeneity in its results, although they were shown in only two studies. Meanwhile, in the measuring tape an average value of 43.84 cm was collected, with values between 23.67 [44] and 64 cm, with minor homogeneity due to assessment in different parts of the body or target populations [50]. In general, all studies collected data during the intervention up to the third month and subsequently did not collect data at the follow-up, except for the studies by Ampomah et al. and Segal et al. which carried out data collection in the first three months.

Table 4 shows data regarding the variable physiological changes of the muscle, in which up to four assessment tools were used: the Fatigue Severity Scale [39], Modified Fatigue Impact Scale [39], Rating Perceived Exertion Scale [51] and accelerometer [47]. For the subvariable fatigue, a disparity was observed between the results, since two scales and a different range of assessment were used in the same study. As for the other subvariables, a direct comparison cannot be made since there are insufficient studies to evaluate it or the results were not collected at some points of the intervention. However, the studies collected results during the first 3 months of intervention and there is only one study that employed a follow-up of a duration equal to 3 months.

For the cardiocirculatory variables shown in Table 5, different measuring instruments were used, such as ultrasound [44], cardiopulmonary exercise test [45], electrocardiogram [52] and electronic manometer [52,53]. For any of the tools observed in Table 5, an average assessment of the results obtained in each of the subvariables cannot be made since, for the most part, all the data were collected in a single study, so it is not possible to directly evaluate. Both studies collected data from the start of the intervention to 3 months, without assessing the post-intervention results by follow-up.

## 4. Discussion

In this review, 20 clinical trials were included, in which the effectiveness of the BFR tool was evaluated together with other exercise methodologies on variables such as strength, muscular structural changes, muscular physiological changes and cardiovascular variables in blood in neuro-musculoskeletal patients. A disparity in the results is found among the observed findings, in which the BFR becomes an alternative tool to high-load exercises in the short and medium term, since the neuromuscular and hormonal reactions caused by the pressure promote the segregation of insulin-like growth factor 1 (IGF-1) and an increase in muscle activation [8]. Some specific aspects are analyzed below.

### 4.1. Strength

For the strength variable (Table 2), heterogeneity is shown in terms of the data collection unit, especially with the dynamometer measurement tool. In the short term, changes in the dynamometer are evidenced in favor of the control groups with 2.3 Nm [38] and the experimental groups with 7.1 Nm [41]. In both studies, the study subjects presented the same pathology and better results when they performed moderate-intensity or low-load exercises. This finding may be since they use different pressure-applying methods with the cuff to the usual protocol, or because patients with knee osteoarthritis present a more complicated clinical picture due to the presence of central sensitization, requiring a multidisciplinary approach [54].

Meanwhile, in one study, changes were observed in favor of the control group for each of the movements in the uninjured leg with average values of 1.9 and 4.1 newton-meter split kilogram (Nm/kg), except for the uncorrected extension, where the BFR group improved 9 Nm/kg more than the control group. For the injured leg, the BFR group presented differences in all movements between 14.6 and 66.2 Nm/kg, although in deficit, the control group found lower differences between both limbs with values between −1.12 and −33.44 Nm/kg [50]. Similarly, patients who received cardiac surgery were able to reduce the loss of strength in the BFR group with an average deficit of 1.2 kg-force for knee extension and fist closure compared to the control group [55]. These between-group differences may be due to the fact that the BFR group uses resistance exercises compared to the control group; therefore, it can be suggested that knee arthroscopy and cardiac surgery patients may benefit from the application of BFR together with resistance exercises; strength, rather than aerobic exercise; or exercise without a pattern of progression [49].

According to the 1RM test, benefits are found for the control group of 2.2 kg [41], while in the 10 RM test, changes are observed for the BFR group in both the uninjured leg with a 0.23 kg match body mass (kg/kg bm) as in the injured leg with 0.305 kg/kg bm [56]. The differences between the patient profiles of each study are minimal; therefore, regardless of using/not using low-load exercise with or without BFR, the same benefits are generated as in the case of the first study. Furthermore, in the second study, the patients improved the strength of both lower limbs, which could be a possible consequence related to the application of greater metabolic stress to replace mechanical stress [7].

The dynamometer tool shows favorable differences for all groups in the medium term, especially for the BFR group, with improvement changes between 11.52% and 26.83% [36,38,] and between −8.57% and 9.09% in the entire knee flexion-extension range on both the unaffected and injured sides, except for the unaffected side at 60 degrees with 14.63% and at 300 degrees with 12.12%, where there was greater improvement in the high-load exercise group [56]. In addition, for patients with cardiac surgery, in the BFR group, it was observed that knee extension strength improved by 36.7% and fist closure by 12.89% [55]. These benefits of the BFR tool explain its usefulness with different exercise methodologies, such as aerobic and strength; in different knee pathologies; or in renal and cardiac pathologies. In addition, pressures between 50% and 80% of the maximum arterial occlusion with the cuff were used at all times, suggesting that the same results would be obtained regardless of this variable [9].

In contrast, there are studies that demonstrate improvements, such as control and experimental improvements, that show changes between −8.1% and 9.34% without statistically significant differences with respect to the BFR group [37,44], with the exception of one study where the control group showed an improvement of 8.4%, i.e., almost double that of the BFR group [42]. The dissonance of this study’s results, with respect to the remaining analyzed studies, may be due to the fact that the BFR group performed the exercise with different parameters to the established protocol for its application, using different maximum repetition percentages for the concentric and eccentric phases of the exercise, as well as the execution time used in each phase of the exercise [13].

For the 1RM tool, the BFR group improved strength by 2.94% [37], although in movements such as knee extension and leg press, the high-load exercise group obtained changes of between 22.85 and 28%; 78% without benefits superior to the BFR group [46,47], which was better in knee extension with a change of 28.78% [48]. Both the BFR and experimental groups improved equally, meaning that high-load and low-load exercise combined with BFR provided the same benefits for subjects with knee pathologies. Greater benefits were found only in one study when applying BFR in older people, which may suggest that this tool is ideal for introducing adequate fatigue that low-load exercise alone cannot provide [57].

In relation to other evaluation tools, changes of 91.15% were found for the injured side and 40.77% for the non-injured side when evaluated with the 10RM after performing high-load exercises [56]. In addition, a difference of 0.2% in the central activation ratio was observed for experimental group 3, which used 70% BFR during the concentric phase and 20% during the eccentric phase of the 1RM in the leg press [37]. The evaluation was not considered statistically significant in either of the two tools with respect to the other groups; however, it indicates that the BFR group improved in the 10RM by inducing metabolic stress [57] and in the case of the central activation ratio, since the combination of eccentric exercise and BFR cuff pressure cause corticalization of the muscle being exercised [58].

Likewise, only one study was found to assess the effects of the BFR tool, where together with the exercise group with moderate loads the same benefits were obtained, although the latter showed a greater difference of 6.5% than the BFR group [53]. These benefits suggest that low-load exercises, together with BFR or moderate loads, can produce the same benefits in patients with chronic kidney disease in phase 2. Therefore, it is noted that applying pressure with the BFR tool obtains good results in terms of strength as a possible consequence of improving patient hemodynamics [40].

Regarding the follow-up, the dynamometer tool demonstrates changes in favor of the control group with 14.3% in subjects with recurrent non-specific lumbar pain [42]. Although there are no major changes compared to the BFR group, it should be noted that this study’s methodology is not well-planned from a treatment point of view; rather, it seeks to observe the cross-transfer effect of the BFR, which does not seem to be provoked but is directly associated with the presence of metabolites [59].

Similarly, the control group shows significant benefits for 4.41% for men with osteoarthritis [41], although the same treatment plan led to better results for women in the BFR group, with a change of 5.38% [40]. In turn, it is extrapolated in the 1RM evaluation, where men in the control group improved by 34.81% and women in the BFR group improved by 17.39%, even 4.92% in 40% of 1RM. These results may suggest that men with osteoarthritis are better adapted to low-load work, while women do so through the use of BFR.

Finally, the BFR group obtained better results in the Manual Muscle Test-8 with an increase of 1.28%, while in the kinetic communicator tool, no changes were found with respect to the evaluation, unlike the control group with negative results [48]. These minor differences shown—with respect to the control group without considerably increasing the benefits—may be due to the use of a random pressure which is not controlled by vascular Doppler where the degree of arterial occlusive pressure can be evaluated, similarly to when the elastic bands are applied [60].

### 4.2. Structural Muscle Changes

Continuing with the structural change variable shown in Table 3, in the short term, improvements were observed for the muscle cross-sectional area by intranuclear magnetic resonance and ultrasound of between −12.65% [57] and −18.75% for the BFR group [35], −13.88% for the control group [49] and 0.03% for the high-load exercise group [56]. This heterogeneity in the results is close to that of the comparative groups; therefore, BFR combined with low-load exercise with passive mobilizations stands as a tool together with high-load exercise to prevent the loss of muscle mass in patients with muscle reconstruction, anterior cruciate ligaments and older adults in a coma [61]. On the other hand, when evaluating muscle thickness using a tape measure, improvements ranging between −5.2% and 6.18% were found [49,50]. In this case, patients with nonreconstructive knee arthroscopy and older adults in a coma could benefit from low-load exercises or passive mobilizations applied together with BFR compared to doing nothing or day-to-day activities as a possible consequence of metabolic stress when submitting [7].

In the medium term, both experimental group 1—which used high-load exercises, with an average of 4.1% [36,46,56] as well as the BFR—and experimental group 3 improved the muscle cross-sectional area, the latter applying restriction of blood flow with different percentages of maximum repetition in the concentric and eccentric phases, with a mean of 1.63% [37,47]. The difference in the results may be due to the use of different evaluation tools such as magnetic resonance imaging, ultrasound and computed tomography, although this difference is significantly small between the experimental and BFR groups. It can also be said that it is a favorable treatment tool for pathologies such as knee or systemic diseases that are both valid in the recovery of muscle mass. This disparity in results was also significantly present in a study where the control group prevented the loss of muscle mass in the erector spinae with −1.7% and the BFR group increased the muscle mass of the quadriceps by 2.9% [42]. In addition, when evaluating muscle thickness with a gold measuring tape, it was found that the control group obtained better results with 0.86% [44]. Therefore, it is suggested that the application of the cuff generates an increase in, or prevention of, loss of muscle mass to the applied area [62].

Regarding the long term, no data is collected. Only in short-term follow-up were increases in muscle mass observed for the BFR groups of 1.92% on average [41,42] and for the control group of 2.5% in the erector spinae [42]. This indicates that post-intervention results continue to be highly beneficial for patients with recurrent nonspecific low back pain and knee osteoarthritis in men, and may be associated with improvements in their quality of life and functionality [63,64].

### 4.3. Physiological Muscle Changes

Within the muscle physiological changes, short-term changes were observed for the fatigue variable in the BFR group of 21.43% with the Modified Fatigue Impact Scale (MFIS) and in the control group of 7.69% with the Fatigue Severity Scale (FSS) [39]. These results confirm that the application of BFR while walking produces the same benefits as walking, although the difference—in favor of the BFR group—may be due to the fact that a lower pressure than usual was applied in the control group [10]. On the other hand, in the effort variable, no differences were found between the BFR groups and high-load exercise for both legs, showing changes in the Rating Perceived of Exertion (RPE) of 13.9% for the BFR group in the injured limb and 14% and 8% for the control group in the injured leg [51]. This finding may indicate that the BFR tool, together with low-load exercises, can achieve the same levels of effort as high-load exercises and may be due to metabolic stress [57].

In relation to the medium term, the experimental group obtained better results than the BFR group in the effort variable, with an average of 15.6% for both members, both healthy and injured [51]. However, the results of the BFR group are similar to those of the experimental group, so it cannot be corroborated that high-load exercises improve fatigue perception more than the low-load exercise group with BFR. An important point in the generation of this greater fatigue may be due to the application of an arterial occlusive pressure of 80%, so that the oxygen supply to the tissues may be lower [61].

In the long term, only one study evaluated the fatigue variable, where significant results were found for the BFR group in the MFIS and FSS, with changes of 21.43% and 5.66%, respectively [39]. In this case, the BFR group may not have generated much fatigue due to the fact that they carried out a different method from the common one, such as intermittent pressure with the cuff, which may reduce this sensation [13].

### 4.4. Hemodynamic Variables and Vascular Caliber

Regarding vascular thickness, all measurements were collected in the medium term. For the vascular thickness of the cephalic vein, the control group obtained better results with the electrocardiogram in measurements of 2, 10 and 20 cm—both for the diameter, with a mean of 8.75%; and for the compliance, with an average of 12.22%. Moreover, the thickness at the cephalic artery level varies minorly, with an average of 9.76% in 2 and 10 cm for the BFR group and in 20 cm for the control group with 10.23% [44]. This difference within the vascular system may be associated with the fact that BFR only acts directly on the arterial system instead of the venous system, hence only improvements in the cephalic artery are observed when used with low-load exercise [65].

Finally, one study assesses the subvariables of respiration, blood pressure and length, showing changes in favor of the group of isometric exercises plus medication intake for the anaerobic threshold of 65.91%, a maximum oxygen volume of 13.43%, ejection fraction of the left ventricle of 6.79% and a final diastolic dimension of the left ventricle of 1.02% [52]. In relation to systolic and diastolic blood pressure, in both isometric exercise groups, moderate loads and BFR obtained similar parameters: around 8.32% on average for systolic blood pressure and 10.31% for diastolic blood pressure [52,53]. However, the group that only received medication obtained greater benefits in the final systolic dimension of the left ventricle with −0.96% [52]. One of the reasons why the isometric exercise group obtained better results than the rest of the groups might be that it is the only group which practiced exercise at a moderate intensity, while the BFR group practiced a protocol of inflating and deflating the cuff without exercising. Therefore, all vascular benefits of the BFR cuff may have been displaced, such as improving blood pressure levels [66].

### 4.5. BFR: Clinical and Trainning Consideration

In view of the clinical applicability of the results observed in the present study, it is important to identify the outcome variable that is intended to be improved when restricting blood flow. In this sense, it was observed how the functional variables undergo a statistically significant improvement in the short and medium term when a moderate blood flow restriction is performed, i.e., 50–60%, while there is more controversy when the blood flow restriction is higher than this percentage or when the pressure exerted is similar for all participants in absolute terms.

On the other hand, in order to obtain structural changes in the muscle in the short-medium term, only significant changes were observed in the diameter of the brachial artery, while in the rest of the structural variables, there is considerable controversy regarding the changes generated, since in some cases it is better than the control group, but equal to or worse than some of the experimental groups that do not have blood flow restriction as a complement to training. In this sense, it may be necessary to carry out longer clinical trials, since the structural changes begin to consolidate in the medium long term, and the improvements observed at the functional level may be the consequence of better efficiency of the motor units, accompanied in the medium term by an increase in muscular vascularization. It would therefore be advisable to design and carry out randomized clinical trials with an analysis of the evolution of the structural characteristics in the medium and long term.

On the other hand, considering the objective of the use of blood flow restriction, i.e., rehabilitation or training, the fact that there is a functional improvement with moderate BFR intensities allows the person being rehabilitated to obtain an increase in function in the short term, which can be key in the first stages of the beginning of recovery since it allows a functional improvement in the short and medium term using moderate blood flow restrictions along with moderate contraction intensities, creating a perfect scenario for those who temporarily experience a reduction in their functional capacity. On the other hand, a longer period of training is required to achieve structural adaptations, so it would be necessary to increase the number of clinical trials aimed at generating structural changes in the muscle. Although it is observed that for training, more intense blood flow restrictions, generating vascular ischemia, or a progressive increase in the intensity of flow restriction seem to have promising results when it comes to training and favoring structural changes.

## 5. Conclusions

The main conclusions that can be reached from carrying out this systematic review are that the application of the BFR technique can achieve benefits in the short and medium term to increase strength, muscle thickness and cardiovascular endurance, as well as improving the physiological level of the cardiovascular system. In addition, BFR combined with low-load exercises also achieves benefits comparable to high-intensity exercises without the application of BFR, benefiting patients who are unable to lift high loads. Some reasons which support these results are the replacement of mechanical stress by metabolic stress and the corticomotor activation of the muscle. However, the results of the included studies are insufficient to indicate a favorable trend from the effects of BFR in the long term and follow-up; therefore, future studies are required to evaluate these periods in order to demonstrate whether its effects are maintained or improved with respect to high-load or aerobic exercises.

## Figures and Tables

**Table 1:** Study characteristics.

Author	Size	Age	Pressure of Cuff	Interventions	Frequency of Sessions	Development Interventions	Pathology
**Ampomah et al.** **[41]**	***n*= 30** **CON: 16** **BFR**: 14	**CON**: 29.9 ± 9.9		**CON**: Isometric ex.	2 ss/w Time: 10 w	**CON**: Workout 25% MVIC, 3 s to task failure (leg extension, plantar flexion and elbow flexion), 30–60 s rest + 3 s (trunk extension), 25% MVIC, 15 reps.	**Recurrent Nonspecific** **Low Back Pain**
		**BFR**: 28.4 ± 9.2	Increase until full restriction and decrease until capillary refill during 2–3 s.	**BFR**: CON + BFR.		**BFR**: CON + BFR.	
**Barbalho et al.** **[34]**	***n*** = 20 * **CON**: 20 **BFR**: 20	**CON**: 66 ± 4.3		**CON**: Passive mobilizations.	1 ss/day Av. d hospital: 11 ± 2.2	**CON**: Passive mobilizations in flexion-extension of lower body, 3 s × 15 reps (2 s in flexion and in extension).	**Elderly Coma Patients**
		**BFR**: 66 ± 4.3	80% arterial systolic	**BFR**: CON + BFR.		**BFR**: CON + BFR.	
**Barbosa et al.** **[43]**	***n*** = 26 **CON**: 14 **BFR**: 12	**CON**: 60.14 ± 10.67		**CON**: Strength training with cuff deflated.	Hospital: 2 ss/w Home: 3 ss/w Time: 8 w	**CON**: Tennis ball squeeze (6 s, 10 reps, 1 min rest, +5 reps/2 w), Elbow flexion (3 s, 10 reps, 1 kg weeks 1 and 2, 2 kg weeks 3 and 4, 3 kg last weeks), Handgrip exercise (3 s, 20 reps, 40% 1RM). 2 min rest/ex.	**Chronic Kidney Disease**
		**BFR**: 61.33 ± 7.82	50% arterial systolic pressure.	**BFR**: CON + BFR		**BFR**: CON + BFR.	
**Ferraz et al.** **[44]**	***n*** = 48 **EG_1_**: 16 **EG_2_**: 16 **BFR**: 16	**EG_1_**: 59.9 ± 4		**EG_1_**: High-intensity workout	20 min/ss 2 ss/w Time: 12 w	**EG_1_**: 1 week (4 s, 10 reps, 50% 1RM), 2 week (4 s, 10 reps, 80% 1RM), 5 week (5 s, 10 reps, 80% 1RM).	**Knee Osteoarthritis**
		**EG_2_**: 60.7 ± 4		**EG_2_**: Low-intensity workout		**EG_2_**: 1 week (4 s, 15 reps, 25% 1RM), 2 week (4 s, 15 reps, 30% 1RM), 5 week (5 s, 15 reps, 30% 1RM)	
		**BFR**: 60.3 ± 3	70% Full BFR	**BFR**: EG_2_ + BFR.		**BFR**: EG_2_ + BFR.	
		**BFR**: 25 ± 2.2	50% Full BFR	**BFR**: External rotation on side-lying + BFR.		**BFR**: CON + BFR (8 min max occlusion).	
**Cardoso et al.** **[42]**	***n*** = 66 **CON**: 22 **EG_1_**: 22 **BFR**: 22	**CON**: 48.2 ± 13.6		**CON**: Usual care	3 ss/w Time: 12 w	**CON**: Usual care pathology in patients.	**End-Stage Renal Disease**
		**EG_1_**: 59.8 ± 16.1		**EG_1_**: Aerobic ex. (bicycle ergometer)		**EG_1_**: 1 Week (60–63% HR, 11–12 Börg scale), 7 Week (64–67% HR, 12–13 Börg scale).	
		**BFR**: 49.4 ± 15.9	50% Full BFR	**BFR**: EG_1_ + BFR		**BFR**: EG_1_ + BFR	
**Chen et al.** **[45]**	***n*** = 55 **EG_1_**: 19 **BFR**: 18 **EG_2_**: 18	**EG_1_**: 62.84 ± 5.54		**EG_1_**: Isometric ex. + EG_2_	**EG_1_**: 2 ss/d, 5 d/w	**EG_1_**: Isometric ex. upper body 40–50% MVC (10 reps-1 min, 1 min rest) + EG_2_.	**Coronary Heart Disease**
		**BFR**: 64.44 ± 8.28	3 min cuff inflation-induced ischemia and 5 min deflation.	**BFR**: Cuff inflation training + EG_2_	**BFR**: 3 ss/d, 5 d/w	**BFR**: 3 min cuff-inflation-induced ischemia and 5 min deflation in both upper limbs alternatively + EG_2_.	
		**EG_2_**: 65.89 ± 5.51		**EG_2_**: Medication	**EG_2_**: Every day	**EG_2_**: Conventional drug treatment.	
					Time: 3 m		
**Corrêa et al.** **[46]**	***n*= 90** **CON**: 30 **EG_1_**: 30 **BFR**: 30	**CON**: 57 ± 6		**CON**: Daily activities	3 ss/w Time: 6 m (3 mesocycles) Mesocycle = 2 m	**CON**: Daily activities	**Stage two of Chronic** **Kidney Disease**
		**EG_1_**: 58 ± 9		**EG_1_**: Resistance training		**EG_1_**: 3 s, 12 rep, 50% 1RM (1° mesocycle); 3 s, 10 rep, 60% 1RM (2° mesocycle); 3 s, 8, 70% 1RM (3° mesocycle)	
		**BFR**: 60 ± 8	50% systolic blood pressure	**BFR**: EG_1_ + BFR		**BFR**: 3 s, 12 rep, 30% 1RM (1° mesocycle); 3 s, 40 rep, 60% 1RM (2° mesocycle); 3 s, 8, 50% 1RM (3° mesocycle) + BFR	
**Curran et al.** **[36]**	***n*** = 34 **EG_1_**: 8 **EG_2_**: 8 **BFR**: 9 **EG_3_**: 9	**EG_1_**: 16.1 ± 2.6		**EG_1_**: Concentrics.	2 ss/w Time: 8 ws	**EG_1_**: 1 s 20% 1RM (PC) + 4 s leg press 70% 1RM concentric—20% 1RM eccentric.	**Anterior Cruciate Ligament Reconstruction**
		**EG_2_**: 18.8 ± 3.9		**EG_2_**: Eccentrics.		**EG_2_**: PC + 4 s leg press 20% 1RM concentric—70% 1RM eccentric.	
		**BFR**: 15.3 ± 0.9	80% Full BFR	**BFR**: Concentrics + BFR		**BFR**: PC + 4 s leg press 70% 1RM concentric—20% 1RM eccentric + BFR.	
		**EG_3_**: 16.0 ± 1.7		**EG_3_**: Eccentrics + BFR		**EG_3_**: PC + 4 s leg press 20% 1RM concentric—70% 1RM eccentric + BFR.	
**Giles et al.** **[35]**	***n*** = 79 **EG_1_**: 39 **BFR**: 40	**EG_1_**: 26.7 ± 5.5		**EG_1_**: Strength training	Trt: 3 ss/w, 8 w (6 individual ss/1–3 w) F/U: 16 w	**EG1**: 5 min bicycle, leg press 0°–60° and knee extension 45°–90°; VAS + 2/10 > ↓ 20% load (PC) + 3 s, 7–10 reps, 70% 1RM, placebo BFR (2 fingers skin/cuff)	**Patellofemoral Pain**
		**BFR**: 28.5 ± 5.2	60% Full BFR	**BFR**: EG_1_ + BFR		**BFR**: PC + 1 set (30 reps or volitive fatigue), 3 s (15 reps), 30% 1RM, 30 s rest.	
**Harper et al.** **[39]**	***n***= 35 **EG_1_**: 19 **BFR**: 16	**EG_1_**: 69.1 ± 7.1		**EG_1_**: Moderate-resistance training	3 ss/w Time: 12 w	**EG_1_**: wmup + leg press, leg extension, leg curl and calf flexion at 60% 1RM + Flexibility-Balance ex.	**Knee Osteoarthritis**
		**BFR**: 67.2 ± 5.2	pressure mm Hg = 0.5 (SBP) + 2(thigh circumference) + 5	**BFR**: EG_1_ + BFR		**BFR**: EG_1_ + BFR 20% 1RM (↓ pression/s).	
**Hughes et al.** **[47]**	***n*** = 28 **EG_1_**: 14 **BFR**: 14	**EG_1_**: 29 ± 7		**EG_1_**: High-resistance training	2 ss/w (48 h rest/ss) Time: 8 w	**EG_1_**: 5 min bicycle no resistance and 10 reps unilateral leg press low load, 5 min rest (PC) + unilateral leg press 70% 1RM, 3 sets, 10 reps, 30 s rest.	**Anterior Cruciate Ligament Reconstruction**
		**BFR**: 29 ± 7	80% Full BFR	**BFR**: EG_1_ + BFR		**BFR**: PC + EG_1_ + BFR 30% 1RM, 4 s (reps: 30, 15, 15, 15).	
**Hughes et al.** **[48]**	***n*** = 28 **EG_1_**: 14 **BFR**: 14	**EG_1_**: 29 ± 7		**EG_1_**: High-resistance training	2 ss/w (48 h rest/ss) Time: 8 w	**EG_1_**: 5 min bicycle no resistance and 10 reps unilateral leg press low load, 5 min rest (PC) + unilateral leg press 70% 1RM, 3 sets, 10 reps, 30 s rest.	**Anterior Cruciate Ligament Reconstruction**
		**BFR**: 29 ± 7	80% Full BFR	**BFR**: EG_1_ + BFR		**BFR**: PC + EG_1_ + BFR 30% 1RM, 4 s (reps: 30, 15, 15, 15).	
**Iversen et al.** **[49]**	***n*** = 24 **CON**: 12 **BFR**: 12	**CON**: 29.8 ± 9.3		**CON**: Quadriceps strength ex.	2 ss/d Time: 12 d	**CON**: 5 s, 20 reps (isometric quadriceps > leg extension over knee roll > straight leg raises).	**Anterior Cruciate Ligament Reconstruction**
		**BFR**: 24.9 ± 7.4	180 mm Hg or maximal pressure tolerable.	**BFR**: CON + BFR		**BFR**: CON + BFR (5 min inflated, 3 min deflated + ex.).	
**Jørgensen et al.** **[50]**	***n*** = 22 **CON**: 11 **BFR**: 11	**CON**: 69.8 ± 4.8		**CON**: No workout.	2 ss/w Time: 12 w F/U: 12 w	CON: Nothing.	**Sporadic Inclusion Body Myositis**
		**BFR**: 68.1 ± 6.4	110 mm Hg	**BFR**: Strength training + BFR.		**BFR**: leg press, knee extension, knee flexion (4 w), calf raise and dorsal flexion. 3 s × 25 reps (9 w: 4 s)	
**Lamberti et al.** ** [38]**	***n*** = 22 **BFR**: 11 **CON**: 11	**BFR**: 54 ± 11		**CON**: Physiotherapy-assisted walking	2 ss/w Time: 6 w F/U: 6 w	**CON**: PC + 40 min physiotherapy-assisted walking, 60 m corridor. Rest: 8/10 RPE on chair.	**Severe Multiple Sclerosis**
		**CON**: 56 ± 10	30% systolic blood pressure	**BFR**: Walking interval-metronome + BFR		**BFR**: 10 min warm up (PC) + 5 cycles (3 reps: 1 min work and 1 min rest. 3 min rest cycle deflated BFR) low-velocity walking (60 steps/min-metronome) + 10 min cool down and stretching CORE (PC).	
**Ogawa et al.** **[51]**	***n*= 21** **CON**: 10 **BFR**: 11	**CON**: 66 ± 8.7		**CON**: Standard cardiac rehab. program	2 ss/w Time: 3 m	**CON**: 30 min aerobic exercise within the level of anaerobic threshold on a cycle ergometer.	**Cardiac open surgery**
		**BFR**: 57 ± 12.2	100–(160–200) mmHg. Increase 20 mmHg/2–3 w.	**BFR**: BFR during Resistance training		**BFR**: Week 1–2: 1 s, 20 rep, 1, 5 s concentric–eccentric (5–10 kg leg extension, 20–30 kg leg press) > 3 s, 30 rep (=weight if Börg < 15 after ex.). Week 3: 3 s, 30 rep, 20–30% 1RM (if Börg < 15 after ex.).	
**Rodrigues et al.** **[52]**	***n*** = 48 **EG_1_**: 16 **BFR**: 16 **CON**: 16	**CON**: 58.1 ± 5.9		**CON**: No workout	2 ss/w Time: 12 w	**CON**: Activities of daily living.	**Rheumatoid Arthritis**
		**EG_1_**: 58.0 ± 6.6		**EG_1_**: High-load workout		**EG_1_**: Bilateral leg press and knee extension. 1 Week: 4 s, 10 reps, 50% 1RM; 2 Week: 4 s, 10 reps, 70% 1RM; 5 Week: 5 s, 10 reps, 70% 1RM.	
		**BFR**: 59.6 ± 3.9	70% Full BFR	**BFR**: Low-load workout + BFR		**BFR**: EG_1_. (1 Week: 4 s, 15 reps, 20% 1RM; 2 Week: 4 s, 15 reps, 30% 1RM; 5 Week: 5 s, 15 reps, 30% 1RM)	
**Segal et al.** **[39]**	***n*** = 42 **CON**: 22 **BFR**: 20	**CON**: 56.1 ± 7.7		**CON**: Low-load workout	3 ss/w Time: 4 w F/U: 3 d	**CON**: Leg press 30% 1RM: 4 s (reps: 30, 15, 15, 15), 30 s rest. Rep: 2 s concentric and 2 s eccentric.	**Knee Osteoarthritis**
		**BFR**: 58.4 ± 8.7	1 Week: 160 mm Hg 2 Week: 180 mm Hg 3 Week: 200 mm Hg	**BFR**: CON + BFR.		**BFR**: CON + BFR.	
**Segal et al.** **[40]**	***n*** = 45 **CON**: 24 **BFR**: 21	**CON**: 54.6 ± 6.9		**CON**: Low-load workout	3 ss/w Time: 4 w F/U: 3 d	**CON**: Leg press 30% 1RM: 4 s (reps: 30, 15, 15, 15), 30 s rest. Rep: 2 s concentric, 2 s eccentric.	**Knee Osteoarthritis**
		**BFR**: 56.1 ± 5.9	1 Week: 160 mm Hg 2 Week: 180 mm Hg 3 Week: 200 mm Hg	**BFR**: CON + BFR.		BFR: CON + BFR.	
**Tennent et al.** **[53]**	***n*** = 24 **CON**: 13 **BFR**: 11	**CON**: 37.0 (32–47)		**CON**: Physiotherapy	12 ss Time: 6 ss	**CON**: Immediate weight loading, immediate formal physiotherapy and no range of motion restrictions.	**Non-Reconstructive Knee Arthroscopy**
		**BFR**: 37.0 (30–46.2)	80% Full BFR	**BFR**: Physiotherapy + (Strength training + BFR)		**BFR**: CON + 4 sets (reps: 30, 15, 15, 15), 30% 1RM, 30 s rest–1 min rest/ex. (leg press, leg extension and reverse press). 5 min max. occlusion/ex.	

**BFR**: Blood flow restriction group; **CON**: Control Group; d: days; **EG_1_**: Experimental group 1; **EG_2_**: Experimental group 2; **EG_3_**: Experimental group 3; h: hours; **HR**: Heart Rate; **kg**: Kilogram; **PC**: Common process; **MVIC**: Maximal voluntary isometric contraction; **reps**: Repetitions; **RM**: Maximal repetition; **RPE**: Rating Perceived Exertion; **s**: Seconds; **ss**: Sessions**VAS**: Visual Analogic Scale; **w**: Weeks. * The author classifies subjects depending on their lower limbs.

**Table 2:** Strength.

Measurement Tool	Article	Group	Baseline	Measurements (SD/CI 95%)			Follow-Up (SD/CI 95%)		
				0–6 Weeks	6–12 Weeks	3–6 Months	1–3 Months	3–6 Months	>6 Months
**Dynamometer**	Ampomah et al. [41] (Nm)	CON	TE: 238.2 ± 97.9 LE: 939.9 ± 301.0	-	8.4% ± 8.2% ^	-	14.3% ± 6.2% ^	-	-
		BFR	TE: 221.2 ± 86.1 LE: 904.1 ± 277.5	-	4.7% ± 7.9% ^	-	−0.1% ± 5.5% ^	-	
	Barbosa et al. [43] (kgf)	CON	24.93 (19.76–30.10)	-	27.29 (22.11–32.46)	-	-	-	-
		BFR	26.83 (21.18–32.48)	-	29.08 (23.86–34.30)	-	-	-	-
	Cardoso et al. [42] (kgf)	CON	53.8 ± 26.6	-	53.9 ± 35.7	-	-	-	-
		EG_1_	70.97 ± 27.12	-	70.28 ± 27.57	-	-	-	-
		BFR	59.72 ± 26.26	-	66.6 ± 32.2	-	-	-	-
	Giles et al. [35] (Nm)	EG_1_	135.1 ± 55.1	-	158.7 ± 57.4	-	-	-	-
		BFR	131.2 ± 61.9	-	166.4 ± 59.4	-	-	-	-
	Harper et al. [37] (Nm)	EG_1_	44.2 *	52.5 ± 3 *	54.75 ± 2.5 *	-	-	-	-
		BFR	44.5 *	50.5 ± 3 *	53 ± 3.5 *	-	-	-	-
	Hughes et al. [47] (N·kg bm)	EG_1_	60°/seg I: 1.8 ± 0.5 * NI: 2.05 ± 0.2 * 150°/seg I: 1.47 ± 0.3 * NI: 1.65 ± 0.3 * 300°/seg I: 1.07 ± 0.23 * NI: 1.65 ± 0.25 *	-	60°/seg I: 1.35 ± 0.5 * NI: 2.35 ± 0.3 * 150°/seg I: 1.25 ± 0.25 * NI: 1.87 ± 0.35 * 300°/seg I: 0.95 ± 0.17 * NI: 1.85 ± 0.35 *	-	-	-	-
		BFR	60°/seg I: 1.75 ± 0.45 * NI: 2.25 ± 0.3 * 150°/seg I: 1.37 ± 0.28 * NI: 1.65 ± 0.2 * 300°/seg I: 1.05 ± 0.2 * NI: 1.65 ± 0.22 *	-	60°/seg I: 1.6 ± 0.5 * NI: 2.45 ± 0.2 * 150°/seg I: 1.42 ± 0.28 * NI: 1.8 ± 0.25 * 300°/seg I: 1.1 ± 0.17 * NI: 1.83 ± 0.28 *	-	-	-	-
	Segal et al. (2015) [39] (Nm)	BFR	169.7 ± 39.0	−0.1 ± 3.3 ¨	-	-	0.4 ± 2.4 ¨^,^^	-	-
		CON	151.9 ± 34.8	7.0 ± 3.0 ¨	-	-	6.7 ± 2.3 ¨^,^^	-	-
	Segal et al. (2015) [40] (Nm)	BFR	1.3 ± 0.3	-	-	-	0.07 ± 0.03 ¨	-	-
		CON	1.3 ± 0.4	-	-	-	0.05 ± 0.03 ¨	-	-
	Tennent et al. [53] (Nm/kg)	BFR	EU (u/i/d): 209.68 (150.13–209.68)/92.81 (68.97–153.41)/106.86 (29.97 a 165.82) FU (u/i/d): 121.21 (95.35–154.16)/91.47 (67.33–108.43)/35.57 (13.38 a 59.26) EC (u/i/d): 215.21 (147.51–251.97)/99.83 (73.83–153.79)/98.34 (29.44 a 145.57) FC (u/i/d): 123.15 (95.5–123.15)/99.83 (79.21–111.34)/31.09 (9.42 a 53.5)	EU (u/i/d): 230.76 (173.07–272.15)/194.59 (132.49–228.51)/34.82 (24.56 a 73.76) FU (u/i/d): 125.69 (111.94–142.73)/131.07 (95.05–140.79)/21.79 (215.99 a 9.57) EC (u/i/d): 225.08 (168.88–285.75)/211.92 (127.48–232.85)/23.01 (29.12 a 64.56) FC (u/i/d): 130.02 (110.75–144.67)/141.68 (110.6–147.06)/22.99 (218.53 a 10.76)	-	-	-	-	-
		CON	EU (u/i/d): 189.81 (185.62–204.15)/124.35 (55.3–156.03)/79.81 (39.16 a 145.27) FU (u/i/d): 124.64 (83.99–126.14)/99.24 (43.34–122.85)/12.85 (214.05 a 46.63) EC (u/i/d): 192.5 (175.76–192.5)/126.74 (100.88–170.75)/68.15 (34.9 a 137.2) FC (u/i/d): 125.09 (84.89–128.38)/105.51 (58.14–129.58)/7.77 (216.44 a 38.65)	EU (u/i/d): 201.76 (169.78–222.98)/181.14 (128.53–217.31)/41.25 (217.93 a 117.47) FU (u/i/d): 130.92 (98.04–139.59)/130.62 (106.78–146.016)/2.39 (215.99 a 9.57) EC (u/i/d): 206.54 (192.87–250.93)/171.57 (120.53–217.9)/42.44 (14.348 a 119.71) FC (u/i/d): 133.91 (97.29–141.17)/132.71 (87.22–142.7)/1.79 (212.2 a 21.89)	-	-	-	-	-
	Curran et al. [36] (Nm)	EG_1_	-	-	ik: −19.2 ± 35.9 is: −13.7 ± 42.6	-	-	-	-
		EG_2_	-	-	ik: −10.8 ± 34.7 is: −10.0 ± 36.3	-	-	-	-
		BFR	-	-	ik: −16.7 ± 21.4 is: −18.0 ± 34.5	-	-	-	-
		EG_3_	-	-	ik: −8.1 ± 16.9 is: −14.6 ± 29.3	-	-	-	-
	Corrêa et al. [46] (kgf)	CON	21.5 ± 6.2	-	-	23.3 ± 4.4	-	-	-
		EG_1_	22.2 ± 5.8	-	-	29.1 ± 3.7	-	-	-
		BFR	24.1 ± 4.9	-	-	30.2 ± 3.2	-	-	-
	Ogawa et al. [51] (kgf)	CON	HG: 31.3 (7.4) KE: 33.5 (10.5)	HG: 28.3 (8.2) KE: 28 (10.4)	HG: 30.7 (6.7) KE: 31.7 (7.48)	-	-	-	-
		BFR	HG: 30.3 (7.5) KE: 30.5 (11.2)	HG: 29.2 (5.2) KE: 29.2 (5.2)	HG: 33.9 (8.5) KE: 41.8 (15.1)	-	-	-	-
**1RM (kg)**	Ferraz et al. [44]	EG_1_	KE: 33 ± 9 * LP: 130 ± 45 *	-	KE: 42.5 ± 9 * LP: 175 ± 50 *	-	-	-	-
		EG_2_	KE: 31.5 ± 11 * LP: 125 ± 40 *	-	KE: 34 ± 10 * LP: 140 ± 45 *	-	-	-	-
		BFR	KE: 33 ± 8.5 * LP: 125 ± 30 *	-	KE: 41 ± 8 * LP: 165 ± 25 *	-	-	-	-
	Rodrigues et al. [52]	EG_1_	KE: 35 ± 12.5 * LP: 113 ± 49.5 *	-	KE: 43 ± 12 * LP: 140 ± 45 *	-	-	-	-
		BFR	KE: 30 ± 12 * LP: 110 ± 30 *	-	KE: 37.5 ± 10.5 * LP: 135 ± 28 *	-	-	-	-
		CON	KE: 33.5 ± 12.5 * LP: 112 ± 35.5 *	-	KE: 33.75 ± 12 * LP: 110 ± 35 *	-	-	-	-
	Segal et al. (2015) [39]	BFR	346.1 ± 95.5 ª	11.3 ± 14.0 ¨	-	-	3.1 ± 0.9 ¨^,^^	-	-
		CON	289.0 ± 48.1 ª	13.5 ± 6.8 ¨	-	-	4.7 ± 1.3 ¨^,^^	-	-
	Segal et al. (2015) [40]	BFR	1RM (kg/kg bm): 2.3 ± 0.6 40% 1RM (W/kg bm): 12.6 ± 2.5	1RM (kg/kg bm): - 40% 1RM (W/kg bm): -	-	-	1RM (kg/kg bm): 0.4 ± 0.3 ¨ 40% 1RM (W/kg bm): 0.62 ± 0.27 ¨	-	-
		CON	1RM (kg/kg bm): 2.1 ± 0.5 40% 1RM (W/kg bm): 11.3 ± 2.9	1RM (kg/kg bm): - 40% 1RM (W/kg bm): -	-	-	1RM (kg/kg bm): 0.2 ± 0.3 ¨ 40% 1RM (W/kg bm): 0.42 ± 0.26 ¨	-	-
	Curran et al. [36]	EG_1_	-	-	2.32 ± 0.90	-	-	-	-
		EG_2_	-	-	1.87 ± 1.00	-	-	-	-
		BFR	-	-	2.94 ± 0.96	-	-	-	-
		EG_3_	-	-	1.94 ± 1.28	-	-	-	-
**10RM** **(kg/kg bm)**	Hughes et al. (2019) [47]	EG_1_	I: 0.475 ± 0.125 * NI: 0.95 ± 0.2 *	I: 0.725 ± 0.925 * NI: 1.13 ± 0.24 *	I: 0.85 ± 0.25 * NI: 1.32 ± 0.25	-	-	-	-
		BFR	I: 0.565 ± 0.125 * NI: 1.03 ± 0.15 *	I: 0.87 ± 0.16 * NI: 1.30 ± 0.17 *	I: 1.08 ± 0.18 * NI: 1.45 ± 0.2 *	-	-	-	-
**CAR**	Curran et al. [36]	EG_1_	-	-	–3.8 ± 11.6	-	-	-	-
		EG_2_	-	-	–3.0 ± 9.2	-	-	-	-
		BFR	-	-	–5.7 ± 10.0	-	-	-	-
		EG_3_	-	-	0.2 ± 7.0	-	-	-	-
**MMT-8**	Jørgensen et al. [50]	CON	68.0 ± 5.5	-	-	-	66.9 ± 6.1	-	-
		BFR	70.3 ± 4.9	-	-	-	71.2 ± 5.4	-	-
**Kinetic Communicator (Nm/kg)**	Jørgensen et al. [50]	CON	0.59 ± 0.57	-	-	-	0.53 ± 0.50	-	-
		BFR	0.62 ± 0.59	-	-	-	0.62 ± 0.55	-	-

**BFR**: Blood flow restriction group; **CAR**: Central activation ratio; **CON**: Control group; **EC**: Extension corrected; **er**: External rotation; **EG_1_**: Experimental group1; **EG_2_**: Experimental group 2; **EG_3_**: Experimental group 3; **EU**: Extension uncorrected; **FC**: Flexion corrected; **FU**: Flexion uncorrected; **HG**: Handgrip; **I**: Injured limb; **ik**: Isokinetic strength; **is**: Maximal isometric voluntary contraction; **KE**: Knee extension; **KF**: Knee flexion; **kg**: Kilogram; **kgf**: Kilogram force; **kg/kg bm**: Kilograms per kilogram body mass; **LP**: Leg press; **MMT-8**: Manual Muscle Test (eight muscles); **NI**: Non-injured limb; **Nm**: Newton meter; **N·kg bm**: Newton per kilogram body mass; **Nm/kg**: Newton meter divided by kilogram; **PTRE**: Peak Torque Right Extension; **PTRF**: Peak Torque Right Flexion; **PTLE**: Peak Torque Left Extension; **PTLF**: Peak Torque Left Flexion; **sp**: supraspinatus; **u/i/d**: uninvolved/involved/deficit; * The author shows the results in figures which t report an estimate value; **-** The author does not report about this information; **¨** The author reports some changes between baseline and measurements or follow-up; **^** The author includes the information in general and percentages; **ª** The author changes the unity of measurement in some evaluations.

**Table 3:** Structural muscle changes.

Measurement Tool		Article	Group	Baseline	Measurements (SD/CI 95%)			Follow-Up (SD/CI 95%)		
					0–6 Weeks	6–12 Weeks	3–6 Months	1–3 Months	3–6 Months	>6 Months
**Cross-** **sectional** **area**	**RMI**	Ampomah et al. [41] (cm^2^)	CON	ES: 22.1 ± 4.3 Q: 57.0 ± 12.6	-	ES: −1.7% ± 2.5% ^ Q: 0.5% ± 1.2% ^	-	ES: 2.5% ± 4.3% ^ Q: 2.3% ± 1.3% ^	-	-
			BFR	ES: 19.8 ± 3.7 Q: 51.5 ± 10.2	-	ES: −3.9% ± 2.7% ^ Q: 2.9% ± 1.05 ^	-	ES: 1.0% ± 4.2% ^ Q: 3.7% ± 1.1% ^	-	-
		Giles et al. [35] (cm)	EG_1_	7.7 ± 1.4	-	7.9 ± 1.2	-	-	-	-
			BFR	7.9 ± 1.3	-	8.0 ± 1.1	-	-	-	-
		Iversen et al. [49] (cm^2^)	CON	40%: 75.4 ± 3.2 50%: 82.8 ± 3.4	40%: 66.1 ± 3.3 50%: 71.3 ± 3.2	-	-	-	-	-
			BFR	40%: 77.5 ± 2.5 50%: 87.0 ± 3.6	40%: 67.7 ± 2.7 50%: 73.9 ± 3.5	-	-	-	-	-
		Segal et al. [40] (cm^3^)	BFR	948.0 ± 71.4	-	-	-	1.3 ± 0.80 ¨	-	-
			CON	1030.8 ± 65.2	-	-	-	0.01 ± 0.73 ¨	-	-
	**Ultrasound**	Barbalho et al. [34] (mm)	CON	11.2 ± 2.7	–2.8 ± 0.7	-	-	-	-	-
			BFR	11.2 ± 2.6	–2.1 ± 0.9	-	-	-	-	-
		Hughes et al. [47] (cm)	EG_1_	-	0.03 ± 0.01	0.12 ± 0.06	-	-	-	-
			BFR	-	0.02 ± 0.01	0.10 ± 0.04	-	-	-	-
		Curran et al. [36] (cm^3^)	EG_1_	-	-	–3.1 ± 3.5	-	-	-	-
			EG_2_	-	-	–2.3 ± 4.3	-	-	-	-
			BFR	-	-	–1.8 ± 2.6	-	-	-	-
			EG_3_	-	-	–1.5 ± 2.4	-	-	-	-
	**TC (mm^2^)**	Ferraz et al. [44]	EG_1_	4700 ± 750 *	-	5150 ± 600 *	-	-	-	-
			EG_2_	4600 ± 950 *	-	4700 ± 950 *	-	-	-	-
			BFR	4650 ± 825 *	-	4950 ± 750 *	-	-	-	-
		Rodrigues et al. [52]	EG_1_	4250 ± 400 *	-	4450 ± 400 *	-	-	-	-
			BFR	4200 ± 225 *	-	4400 ± 300 *	-	-	-	-
			CON	4350 ± 450 *	-	4375 ± 425 *	-	-	-	-
**Muscular** **thickness**	**Measuring** **tape (cm)**	Barbalho et al. [34]	CON	48.2 ± 2.5	–3.6 ± 1.3 ¨	-	-	-	-	-
			BFR	48.1 ± 2.9	–2.5 ± 1.1 ¨	-	-	-	-	-
		Barbosa et al. [43]	CON	25.62 (23.67–27.56)	-	25.84 (24.11–27.57)	-	-	-	-
			BFR	26.27 (24.87–27.67)	-	26.49 (25.13–27.85)	-	-	-	-
		Tennent et al. [53]	CON	6 cm-p (u/i): 50.00 (44–52)/49.00 (45.5–51) 16 cm-p (u/i): 59.50 (53–62)/60.00 (54–61)	6 cm-p (i): 50.00 (45.5–50.5) 16 cm-p (i): 60.00 (54–61)	-	-	-	-	-
			BFR	6 cm-p (u/i): 46.50 (43–53.25)/44.50 (42.3–50.5) 16 cm-p (u/i): 58.00 (51.4–63.3)/54.50 (50.3–61.4)	6 cm-p (i): 47.25 (45.5–53.6) 16 cm-p (i): 57.50 (51.6–64)	-	-	-	-	-

**40%**: 40% length of femur from distal to proximal in lateral-side knee; **50%**: 50% length of femur from distal to proximal in lateral-side knee; **BFR**: Blood flow restriction group; **cm**: Centimeters; **cm^2^**: Centimeters squared; **CON**: Control group; **cm-p**: Centimeters of patella superior border; **CSA**: Cross-sectional area; **EG_1_**: Experimental group 1; **EG_2_**: Experimental group 2; **EG_3_**: Experimental group 3; **ES**: Spinal erectors; **mm**: Millimeters; **mm^2^**: Millimeters squared; **Q**: Quadriceps; **RMI**: Intranuclear resonance magnetic; **TC**: Computerized tomography; **u/i**: uninvolved/involved; **W/kg bm**: Weight per kilogram of body mass; * The author shows the results in figures which report an estimate value; - The author does not report about this information; **^** The author includes the information in general and percentages; **¨** The author reports some changes between baseline and measurements or follow-up.

**Table 4:** Physiological muscle changes.

Measurement Tool		Article	Group	Baseline	Measurements (SD/CI 95%)			Follow-Up (SD/CI 95%)		
					0–6 Weeks	6–12 Weeks	3–6 Months	1–3 Months	3–6 Months	>6 Months
**Fatigue**	**MFIS (0–84)**	Lamberti et al. [38]	BFR	42 (32–52)	33 (20–46)	-	-	33 (21–45)	-	-
			CON	33 (25–41)	24 (14–33)	-	-	28 (17–38)	-	-
	**FSS (9–63)**	Lamberti et al. [38]	BFR	5.3 (4.9–5.8)	5.1 (4.4–5.9)	-	-	5.0 (4.4–5.6)	-	-
			CON	5.2 (4.7–5.6)	4.8 (4.2–5.3)	-	-	5.0 (4.6–5.5)	-	-
**Exertion**	**RPE**	Hughes et al. [38]	EG_1_	-	13.8 ± 2.1 (I)/14.8 ± 2 (NI) *	15.5 ± 2.3 (I)/15.7 ± 2.2 (NI) *	-	-	-	-
			BFR	-	13.9 ± 2 (I)/14.75 ± 2 (NI) *	14.5 ± 2 (I)/15.3 ± 2.2 (NI) *	-	-	-	-
**Time**	**Accelerometer (min/day)**	Rodrigues et al. [52]	EG_1_	S: 495.5 ± 93.1	-	-	-	-	-	-
				L: 368.4 ± 76.7		-				
				MV: 16.4 ± 14.1		-				
			BFR	S: 702.6 ± 246.1	-	-	-	-	-	-
				L: 317.9 ± 98.0		-				
				MV: 16.8 ± 13.8		-				
			CON	S: 637.2 ± 263.0	-	-	-	-	-	-
				L: 365.7 ± 96.5		-				
				MV: 21.4 ± 15.2		-				

**BFR**: Blood flow restriction group; **EG_1_**: Experimental group 1; **FSS**: Fatigue severity scale; **I**: Injured limb; **L**: Light physical activity; **min/day**: Minutes per day; **MFIS**: Modified fatigue impact scale; **MV**: Moderate to vigorous activity; **NI**: Non-injured limb; **RPE**: Rating Perceived Exertion Scale; **S**: Sedentary activity; * The author shows the results in figures which they report an estimate value.

**Table 5:** Blood/cardiocirculatory outcomes.

Measurement Tool		Article	Group			Baseline	Measurements (SD/CI 95%)			Follow-Up (SD/CI 95%)		
							0–6 Weeks	6–12 Weeks	3–6 Months	1–3 Months	3–6 Months	>6 Months
**Vascular** **thickness**	**Ultrasound (mm)**	Barbosa et al. [43]	Cephalic vein	2 cm	CON	D_1_: 2.71 (2.39–3.02)	-	D_1_: 2.94 (2.65–3.23)	-	-	-	-
						D_2_: 2.62 (2.26–2.98)		D_2_: 2.97 (2.74–3.20)				
					BFR	D_1_: 2.50 (2.05–2.95)	-	D_1_: 2.70 (2.30–3.11)	-	-	-	-
						D_2_: 2.55 (2.14–2.97)		D_2_: 2.69 (2.34–3.04)				
				10 cm	CON	D_1_: 3.06 (2.61–3.51)	-	D_1_: 3.45 (3.01–3.88)	-	-	-	-
						D_2_: 3.01 (2.36–3.66)		D_2_: 3.41 (2.94–3.88)				
					BFR	D_1_: 2.74 (2.16–3.32)	-	D_1_: 2.90 (2.30–3.50)	-	-	-	-
						D_2_: 2.69 (2.00–3.15)		D_2_: 2.81 (2.35–3.27)				
				20 cm	CON	D_1_: 3.40 (2.95–3.86)	-	D_1_: 3.57 (3.08–4.05)	-	-	-	-
						D_2_: 3.20 (2.77–3.62)		D_2_: 3.52 (3.12–3.93)				
					BFR	D_1_: 2.95 (2.28–3.62)	-	D_1_: 3.10 (2.46–3.74)	-	-	-	-
						D_2_: 3.05 (2.41–3.70)		D_2_: 2.90 (2.26–3.53)				
			Radial artery	2 cm	CON	D_1_: 2.82 (2.55–3.10)	-	D_1_: 2.95 (2.64–3.26)	-	-	-	-
					BFR	D_1_: 2.53 (2.21–2.85)	-	D_1_: 2.77 (2.50–3.04)	-	-	-	-
				10 cm	CON	D_1_: 2.90 (2.62–3.17)	-	D_1_: 3.02 (2.75–3.29)	-	-	-	-
					BFR	D_1_: 2.59 (2.21–2.96)	-	D_1_: 2.85 (2.42–3.29)	-	-	-	-
				20 cm	CON	D_1_: 3.03 (2.61–3.45)	-	D_1_: 3.34 (3.05–3.63)	-	-	-	-
					BFR	D_1_: 2.93 (2.46–3.39)	-	D_1_: 3.11 (2.69–3.53)	-	-	-	-
**Breathing**	**CPET (Anaerobic umbral: mL/kg/min)**	Chen et al. [45]	EG_1_			11.15 ± 2.64	-	18.5 ± 3.5 ^	-	-	-	-
			BFR			11.26 ± 3.16	-	15 ± 2 ^	-	-	-	-
			EG_2_			11.86 ± 2.57	-	11.75 ± 2 ^	-	-	-	-
	**CPET (VO** **_2_** **max: mL/kg/min)**	Chen et al. [45]	EG_1_			33.50 ± 4.28	-	38 ± 6 ^	-	-	-	-
			BFR			32.18 ± 5.39	-	35.5 ± 6.5 ^	-	-	-	-
			EG_2_			32.76 ± 5.92	-	32.7 ± 5.9 ^	-	-	-	-
**Blood** **pressure**	**SBP (mmHg)**	Chen et al. [45]	EG_1_			143.32 ± 7.48	-	133 ± 7.5 ^	-	-	-	-
			BFR			143.94 ± 9.55	-	140 ± 4.25 ^	-	-	-	-
			EG_2_			145.78 ± 7.73	-	144 ± 6 ^	-	-	-	-
		Corrêa et al. [46]	CON			142.7 ± 10.7	-	-	141.7 ± 10.1	-	-	-
			EG_1_			143.0 ± 10.1	-	-	129.5 ± 10.6	-	-	-
			BFR			141.4 ± 10.2	-	-	128.2 ± 10	-	-	-
	**DBP (mmHg)**	Chen et al. [45]	EG_1_			82.63 ± 7.65	-	76 ± 5.5 ^	-	-	-	-
			BFR			83.50 ± 7.12	-	81 ± 7 ^	-	-	-	-
			EG_2_			83.22 ± 6.53	-	84 ± 6 ^	-	-	-	-
		Corrêa et al. [46]	CON			92.4 ± 9.8	-	-	92.6 ± 11	-	-	-
			EG_1_			93.8 ± 10.3	-	-	82.2 ± 11.2	-	-	-
			BFR			94.4 ± 9.5	-	-	82.5 ± 12.5	-	-	-
**Length**	**ECG (LVEF: %)**	Chen et al. [45]	EG_1_			54.21 ± 7.38	-	61 ± 4.5 ^	-	-	-	-
			BFR			53.39 ± 7.41	-	57.5 ± 5.5 ^	-	-		-
			EG_2_			51.44 ± 7.60	-	52 ± 7.25 ^	-	-	-	-
	**ECG (LVEDD: mm)**	Chen et al. [45]	EG_1_			47.53 ± 7.31	-	43.75 ± 6.5 ^	-	-	-	-
			BFR			48.44 ± 8.46	-	45.5 ± 7.75 ^	-	-	-	-
			EG_2_			50.89 ± 7.45	-	50.4 ± 7.5 ^	-	-	-	-
	**ECG (LVESD: mm)**	Chen et al. [45]	EG_1_			35.68 ± 6.54	-	29.75 ± 5.25 ^	-	-	-	-
			BFR			36.22 ± 6.81	-	33.5 ± 6.5 ^	-	-	-	-
			EG_2_			38.11 ± 7.11	-	38.5 ± 6.25 ^	-	-	-	-

**BFR**: Blood flow restriction group; **cm**: Centimeters; **CON**: Control group; **CPET**: Cardiopulmonary exercise test; **EG_1_**: Experimental group 1; **EG_2_**: Experimental group 2; **D_1_**: Diameter; **D_2_**: Distensibility; **ECG**: Electrocardiogram; **LVEDD**: Left ventricular end-diastolic dimension; **LVESD**: Left ventricular end-systolic dimension; **LVEF**: left ventricular ejection fraction; **mm**: Millimeters; **mL/kg/min**: millimeters per kilogram per minute; **VO_2_ max**: Maximum volume of oxygen; * The author shows the results in figures which they report an estimate value; **^** The author includes the information in general and percentages.
